# Q&A: What is biodiversity?

**DOI:** 10.1186/1741-7007-8-145

**Published:** 2010-12-15

**Authors:** Anne E Magurran

**Affiliations:** 1 School of Biology, University of St Andrews, St Andrews, Fife, KY16 8LB, UK

## 2010 is the UN's International Year of Biodiversity. What's that all about?

The UN declared 2010 the International Year of Biodiversity to celebrate life on earth, and to highlight attempts to safeguard this diversity. There have been numerous biodiversity meetings and related activities during the year, and growing awareness that the diversity of life on earth is under threat as never before.

## What is biodiversity?

Biodiversity is simply the variety of life. This can mean anything from the microbes in a few grams of soil to all the organisms that inhabit the earth. In practice, to assess how much diversity we have and what it does, we need to be more specific about the aspect of biodiversity we are concerned with, and the area and time frame over which we want to measure it. For example, we might consider the types and relative abundances of species of trees in a forest, or the genetic diversity associated with the individuals of those species, or even how the number and composition of forests across a biogeographic region have changed over the past century. This hierarchy of organizational levels is implicit in the definition developed by the UN in their Convention on Biological Diversity, which states that biological diversity is 'the variability among living organisms from all sources, including, *inter alia*, terrestrial, marine, and other aquatic ecosystems, and the ecological complexes of which they are part: this includes diversity within species, between species and of ecosystems'.

## You mentioned the spatial and temporal context of biodiversity. What do you mean by this?

How much biodiversity we see, such as the number of bird species recorded in a wetland, increases both as a larger area is surveyed, and as the same area is surveyed for a longer time. Thus, if we were to watch the same lake over several years, we would find that some new species would colonize, others would vanish from the area, and there would be occasional unexpected species appearing, perhaps diverted away from their normal migration route by unusual weather conditions. These species-area and species-time relationships are well-known 'laws' of ecology, yet are sometimes overlooked when investigators or conservationists are making comparisons between sites or deciding which places should be designated as nature reserves.

## Why should we care about biodiversity?

As Charles Elton explained in his book *The Ecology of Invasions by Animals and Plants*, over half a century ago now, there are three main arguments for conserving biodiversity. The first is that other species have the right to exist on the planet, and therefore it would be unethical to take actions that might cause their extinction. The second is the aesthetic value of biodiversity. Humans derive pleasure and a sense of wellbeing from wild nature. This quality of biodiversity, while implicitly recognized by most people, has been attributed by EO Wilson to a deep-seated evolutionary need to live in a favorable environment. The third reason proffered for biodiversity conservation - and the one that receives most attention - is the utilitarian argument. The reasoning here is that biodiversity underpins many goods and services that we depend on. These include wild harvests (such as fish), pollination, carbon capture (for example, peat bogs and rain forests store carbon that would otherwise be released to the atmosphere), regulation of drainage patterns and climate, soil formation, flood defenses (including the mangrove swamps that help protect low lying areas against tsunamis) and the genetic resources used in agriculture and medicine.

## How long have researchers been interested in biodiversity?

Biodiversity is something that has probably always been recognized and appreciated. Our ancestors, after all, will have been aware of those places where there was a rich abundance of food, and those that harbored dangerous predators. However, the scientific study of biodiversity really began when Carl Linneaus put together the first systematic catalogue of life on earth. His book, *Species plantarum*, published in 1753, classified and described around 7,000 species and developed the system of taxonomy that we still use today. This was also the beginning of the great age of scientific exploration. Alexander von Humbolt traveled to South America between 1799 and 1804 and made many observations about biological diversity, most famously that there is a latitudinal gradient of richness, with tropical habitats having many more species than temperate habitats, which are, in turn, more diverse than boreal regions. Joseph Banks, another early explorer, commented on the differences in variety of species of flora at different localities. Charles Darwin's voyage on *The Beagle *is well known for the role it played in shaping his thinking about evolution. Less well appreciated is that the *Origin of Species *contains many insightful reflections on patterns of biological diversity, and Darwin was one of the first researchers (along with Audubon) to comment on the fact that ecological communities are invariably composed of a few common and many rare species. Darwin's contemporaries, including Wallace and Bates, also wrote extensively about patterns of biological diversity. Nonetheless, it was not until the 20th century that ecologists began to develop new statistical methods for quantifying biodiversity.

## How is biodiversity measured?

**Figure 1:**
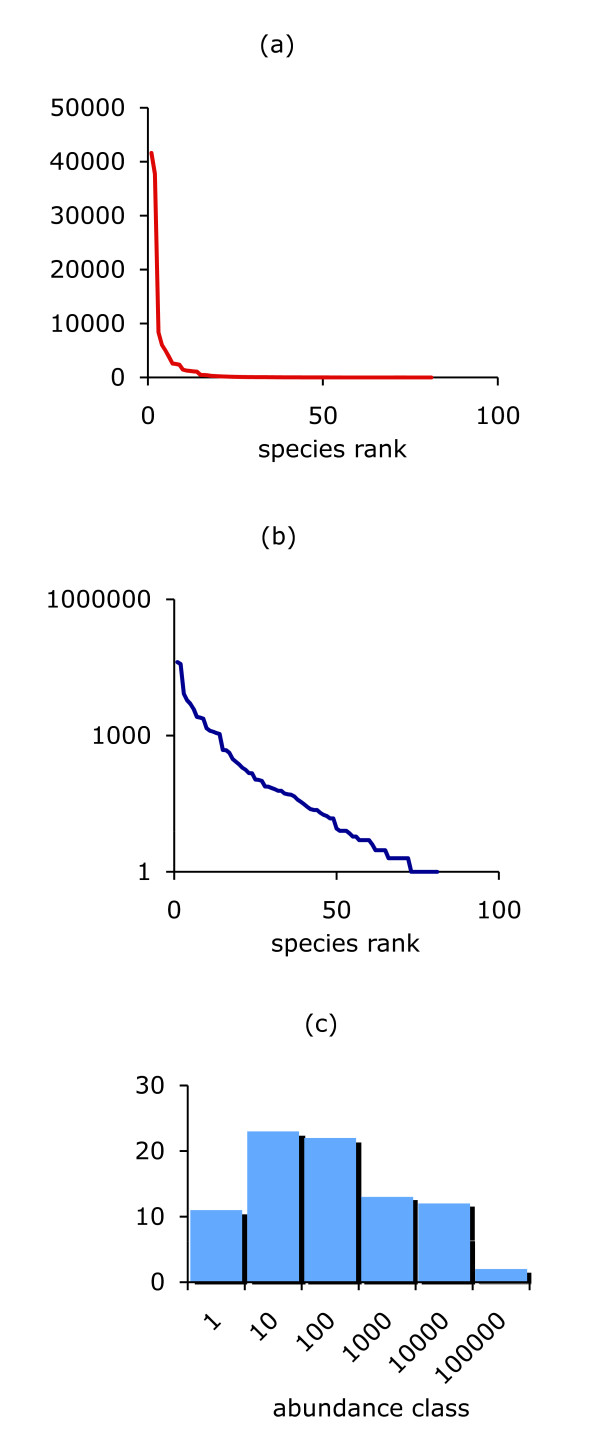
**There are many more rare species than common ones in ecological assemblages**. These data, representing the numbers of individuals in the 81 species making up a 25 year time series of estuarine fish in the UK's Bristol Channel, were collected by PA Henderson. **(a) **The data plotted on an arithmetic scale to emphasize the handful of exceptionally abundant species; **(b) **the same data, with the abundance now plotted on a logarithmic scale (this type of graph is usually called a rank abundance plot); and **(c) **a histogram showing the frequency of species in logarithmic abundance classes. The plot in (c), which was introduced by Frank Preston, illustrates the log normal pattern of diversity often seen in ecological assemblages. These different methods of plotting species abundance data highlight contrasting aspects of the assemblage, but they all underline the universal pattern of a few abundant and many rare species.

Sir Ronald Fisher, the famous statistician, was one of the first researchers to recognize that the characteristic pattern of commonness and rarity of species could be described mathematically. The resulting species abundance distribution, the 'log series model', is associated with an index of diversity known as Fisher's α. Other models and measures were soon developed, and two enduring approaches are Preston's log normal distribution of species abundances and the Shannon diversity index. There are now a great many competing methods of measuring biodiversity. In essence, the approaches either describe the entire distribution of species abundance (Figure 1), or provide some metric that quantifies the richness of the sample or assemblage (richness being the number of species present), or develop a statistic that takes account of the evenness of the species abundances. Although formulated in the context of species, these measures can also be adapted to measure diversity at other organizational levels, including genes and traits.

A complementary approach to biodiversity assessment is to ask how different two or more localities, or indeed time periods, are in terms of their species composition. Thus, Robert Whittaker distinguished between α diversity - that is, the biodiversity at a defined place and time (and he deliberately used the term α diversity to link with Fisher's concept of a diversity index) - and β diversity, which is a measure of how much variation there is in the number and identity of species across space and/or time. A landscape will be most diverse if it supports ecological communities with high α diversity and also high β diversity.

## How many species are there?

An obvious question, but one that is very difficult to answer! As Bob May has pointed out, only around 1.7 million species have so far been named. However, even this total is beset with difficulties: some of these will be duplications (known as synonomies) and there are also many gaps in the record. What we do know is that vertebrates (especially mammals and birds) are reasonably well documented, but invertebrates (including most insects) are not. JBS Haldane, when asked what creation revealed about the nature of the creator, gave the celebrated response 'an inordinate fondness for beetles'. It is no surprise, therefore, to learn that beetles remain a very poorly documented group. May estimates that there are probably between 5 and 10 million species, but also notes that the number could lie anywhere between 3 and 100 million. And that's before we start thinking about microbes.

## How do we know that biodiversity is being lost, given the great uncertainty about species numbers?

Although it can be difficult to put precise numbers on how many, and which, species are being lost, there is no doubt that biodiversity is declining. This can be deduced in a number of ways. First, we know that habitat is being lost. Because of the well-known relationship between the area of a habitat and the number of species it supports, it is possible to work out how a reduction in area will translate into a reduction in biodiversity. Second, it is not just α diversity that is being lost; β diversity is also diminishing. This is because the same exotic species are being introduced into many different areas with the result that the ecological communities become more similar or 'homogenized'. Third, researchers have developed a series of indicators to track the decline in biodiversity. One example is the 'Living Planet Index', which uses trends in vertebrate species diversity as an indicator of global diversity. Other approaches involve long-term monitoring and use data compiled for the International Union for Conservation of Nature's red list to track species status. Finally, meta-analyses can pull together data from a range of sources to produce an overview of change and biodiversity loss.

## Why is biodiversity being lost?

Jared Diamond dubbed the reasons for biodiversity loss as the 'evil quartet' - habitat loss, over-exploitation of species, invasive species together with the exotic diseases they introduce, and the breakdown of ecological networks. Habitat degradation is perhaps the most obvious of these as the growing human population, and the growing demands for consumer products, place ever greater strains on the environment.

## What are the 2010 and 2020 biodiversity targets?

The Convention for Biological Diversity (CBD) set itself the target of reducing the rate of biodiversity loss by 2010. It is now generally agreed that this target has not been met. Recently, the CBD convened at Nagoya in Japan and established a new set of targets for 2020. There are 20 of these and they address a range of biosecurity, conservation and long-term scientific and socio-economic goals. Although the 2020 targets are more focused, and thus probably more achievable, than the 2010 target, not everyone is convinced that biodiversity is any better protected. George Monbiot is one environmental commentator who remains to be persuaded.

## What can I do to help conserve biodiversity?

While dramatic events - including the threatened extinction of charismatic species such as the tiger or giant panda, or the loss of swathes of habitat, such as the felling of tropical forest in Southeast Asia to make way for palm oil plantations - make headline news, it is the accumulation of many small decisions that lead to the steady erosion of biodiversity. Roger Deakin, writing about the loss of a small spinney in his 2008 book *Notes from Walnut Tree Farm*, describes how the bulldozed mud 'smothered all the wild flowers, hibernating bumblebees etc., especially an extensive bed of violets that grew, with the assertive dog's mercury and lords and ladies, under the shade of the trees'. The choices we make about where we build our houses and golf courses, the food and consumer goods we buy in the shops, how many children we have, and the extent to which we hold our governments to account on their promises to develop environmentally friendly policies collectively determine the fate of biodiversity.

Where can I find out more?

Books

1. Magurran AE, McGill BJ: *Biological Diversity: Frontiers in Measurement and Assessment*. Oxford: Oxford University Press; 2011.

2. May RM: **Unanswered questions and why they matter**. In *Theoretical Ecology: Principles and Applications*. 3rd edition. Edited by May RM, McLean AR. Oxford: Oxford University Press; 2007:205-215.

Articles

1. Perrings C, Naeem S, Ahrestani F, Bunker DE, Burkill P, Canziani G, Elmqvist T, Ferrati R, Fuhrman J, Jaksic F, Kawabata Z, Kinzig A, Mace GM, Milano F, Mooney H, Prieur-Richard AH, Tschirhart J, Weisser W: **Ecosystem services for 2020**. *Science *2010, **330**:323-324.

2. Butchart SH, Walpole M, Collen B, van Strien A, Scharlemann JP, Almond RE, Baillie JE, Bomhard B, Brown C, Bruno J, Carpenter KE, Carr GM, Chanson J, Chenery AM, Csirke J, Davidson NC, Dentener F, Foster M, Galli A, Galloway JN, Genovesi P, Gregory RD, Hockings M, Kapos V, Lamarque JF, Leverington F, Loh J, McGeoch MA, McRae L, Minasyan A, *et al*: **Global biodiversity: indicators of recent declines**. *Science *2010, **328**:1164-1168.

3. Boitani L, Cowling RM, Dublin HT, Mace GM, Parrish J, Possingham HP, Pressey RL, Rondinini C, Wilson KA: **Change the IUCN protected area categories to reflect biodiversity outcomes**. *PLoS Biol *2008, **6**:e66.

4. Magurran AE, Baillie SR, Buckland ST, Dick JMP, Elston DA, Scott EM, Smith RI, Somerfield PJ, Watt AD: **Long-term datasets in biodiversity research and monitoring: assessing change in ecological communities through time**. *Trends Ecol Evol *2010, **25**:574-582.

5. Loh J, Green RE, Ricketts T, Lamoreux J, Jenkins M, Kapos V, Randers J: **The Living Planet Index: using species population time series to track trends in biodiversity**. *Philos Trans R Soc Lond B Biol Sci *2005, **360**:289-295.

6. Rahel FJ: **Homogenization of fish faunas across the United States**. *Science *2000, **288**:854.

7. Jackson JBC: **The future of the oceans past**. *Philos Trans R Soc B Lond Biol Sci *2010, **365**:3765-3778.

Links

1. **United Nations International Year of Biodiversity **http://www.cbd.int/2010/welcome/

2. **George Monbiot: A Ghost Agreement **http://www.monbiot.com/archives/2010/11/01/a-ghost-agreement/

